# Comprehensive transcriptome data for endemic Schizothoracinae fish in the Tibetan Plateau

**DOI:** 10.1038/s41597-020-0361-6

**Published:** 2020-01-21

**Authors:** Chaowei Zhou, Shijun Xiao, Yanchao Liu, Zhenbo Mou, Jianshe Zhou, Yingzi Pan, Chi Zhang, Jiu Wang, Xingxing Deng, Ming Zou, Haiping Liu

**Affiliations:** 1 Institute of Fisheries Science, Tibet Academy of Agricultural and Animal Husbandry Sciences, Lhasa, 850002 China; 2 Departments of Aquaculture, College of Animal Science, Southwest University, Chongqing, 402460 China; 3 School of Computer Science and Technology, Wuhan University of Technology, Wuhan, Hubei 430000 China

**Keywords:** RNA sequencing, Ichthyology, Transcriptomics

## Abstract

The Schizothoracinae fishes, endemic species in the Tibetan Plateau, are considered as ideal models for highland adaptation and speciation investigation. Despite several transcriptome studies for highland fishes have been reported before, the transcriptome information of Schizothoracinae is still lacking. To obtain comprehensive transcriptome data for Schizothoracinae, the transcriptome of a total of 183 samples from 14 representative Schizothoracinae species, were sequenced and *de novo* assembled. As a result, about 1,363 Gb transcriptome clean data was obtained. After the assembly, we obtain 76,602–154,860 unigenes for each species with sequence N50 length of 1,564–2,143 bp. More than half of the unigenes were functionally annotated by public databases. The Schizothoracinae fishes in this work exhibited diversified ecological distributions, phenotype characters and feeding habits; therefore, the comprehensive transcriptome data of those species provided valuable information for the environmental adaptation and speciation of Schizothoracinae in the Tibetan Plateau.

## Abstract (summary)

**Table:** 

Measurement(s)	RNA • transcriptome • sequence_assembly • sequence feature annotation
Technology Type(s)	RNA sequencing • sequence assembly process • sequence annotation
Sample Characteristic - Organism	Schizothorax oconnori • Schizothorax lissolabiata • Schizopyge nukiangensis • Schizothorax plagiostomus • Schizothorax labiatus • Schizothorax davidi • Ptychobarbus kaznakovi • Gymnocypris namensis • Gymnocypris przewalskii • Gymnocypris eckloni • Gymnocypris selincuoensis • Schizopygopsis younghusbandi • Schizopygopsis pylzovi • Platypharodon extremus • Oxygymnocypris stewartii
Sample Characteristic - Environment	lake • drainage basin
Sample Characteristic - Location	Tibetan Plateau

Machine-accessible metadata file describing the reported data: 10.6084/m9.figshare.11475294

## Background & Summary

The Tibetan Plateau, the world’s largest and highest plateau, has unique geographical and climatic characteristics, such as the high altitude, dramatic difference in day and night temperature, strong solar radiation^1^. Due to the special geographical environment, many highland species that are distributed in and around the Tibetan Plateau have gradually formed unique characteristics to tolerate harsh living conditions during the long-term evolution^2^. The Schizothoracinae fishes, members of family Cyprinidae, are endemic to Asian highlands including 15 genera and ca. 100 species^3^. In China, more than 70 species, account for over 80% of the world’s Schizothoracine fishes, are mainly distributed in lakes and rivers of the Tibetan Plateau and adjacent areas^4^. According to the morphological characteristics, the Schizothoracine fishes can be divided into three groups: the primitive group, the specialized group and the highly specialized group^5^. Several researches on the morphology, archaeology and molecular biology of Schizothoracine fishes on the Tibetan Plateau have shown that there is close correlation between the species diversity and the uplift of the Tibetan Plateau^6,7^ and the morphological traits of Schizothoracine fishes is related with specific periods of geological evolution of the Tibetan Plateau such as pharyngeal teeth, scales and whiskers^5^. Therefore, the Schizothoracine fishes are considered as good model species for the investigations on highland adaptation and speciation. More genomic and transcriptome data are required to decipher the relationship of the speciation and the uplift of the Tibetan Plateau for the Schizothoracine fishes.

**Fig. 1:**
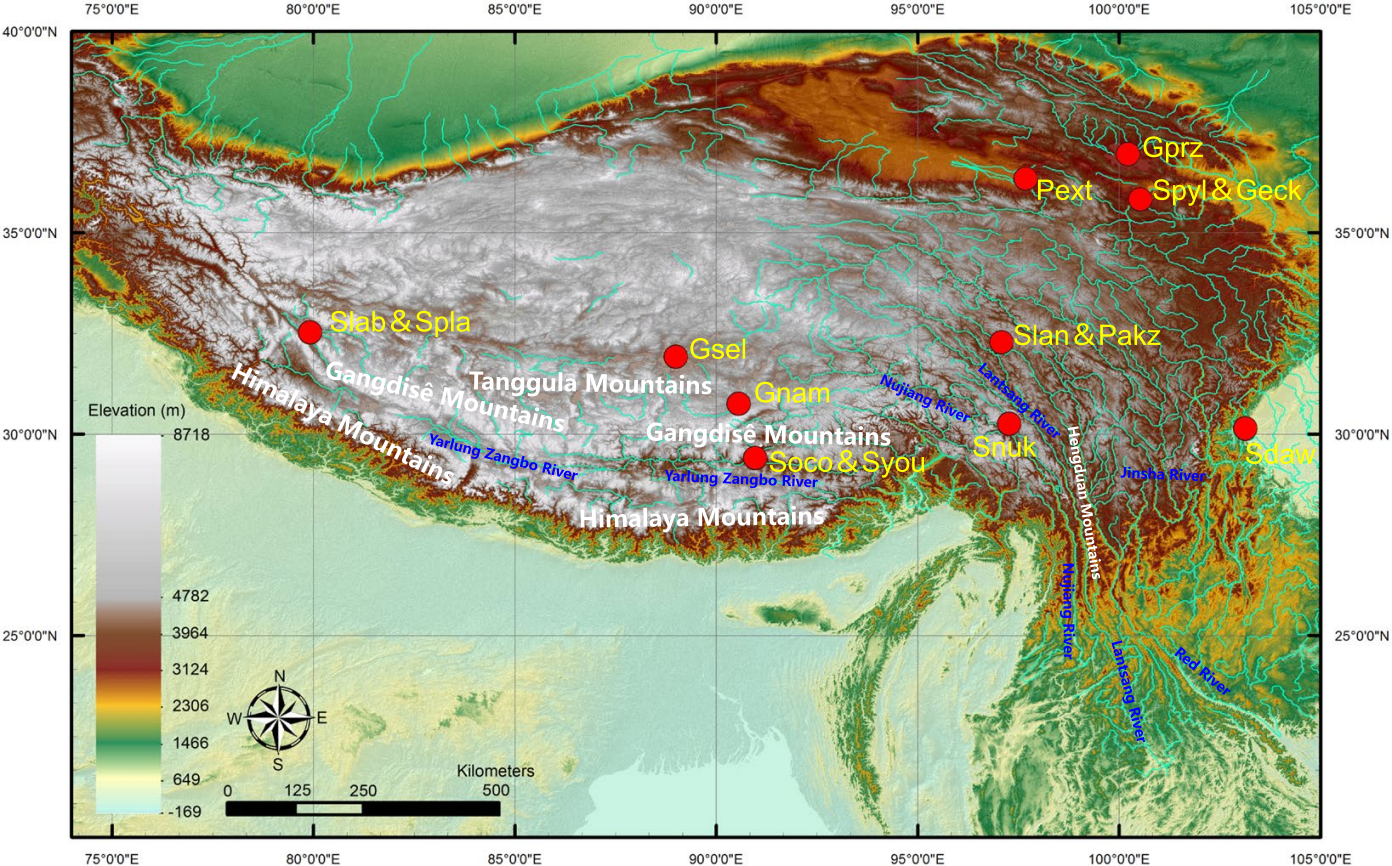
Sample sites of 14 *Schizothoracine* species in our study. The abbreviations of species were identical with those in Table 1. The altitude was represented by the color bar from white (high alititude) to green (low altitude).

Recent advances in sequencing technologies have offered the opportunity to obtain the genomes of numerous highland animals, enabling us to better understand the adaptive evolution mechanism of highland fish species. So far, the vast majority of the genome researches on the environmental adaptation were performed on highland terrestrial animal (e.g., yak^8^ and Tibetan antelope^9^). Few study was reported on highland fish, especially for Schizothoracinae fishes. One of the major reasons was the complexity of the genome, such as high content of repeats and polyploidy^10^. Transcriptome sequencing is a good choice to construct the sequence dataset for transcribed genes in many polyploidy cases^11^. Despite several transcriptome analyses on highland adaptation have reported in Schizothoracine fishes before^12–16^, the species and tissues used for transcriptome sequencing were still limited. There is a great demand for more transcriptome sequencing data for the adaptation and evolution of Schizothoracine fishes in the Tibetan Plateau. In this work, we obtained and released a total of ∼1.36 Tb of high-quality transcriptome data for 183 samples of 14 representative Schizothoracine fish covering 5 genera from 6 drainage systems and 3 lakes in the Tibetan Plateau (Tables 1, 2 and Fig. 1). The distribution, ecological position and phenotype difference making the transcriptome of those Schizothoracine species invaluable genetic resources for the adaptation and speciation of endemic fish in the Tibetan Plateau.

**Table 1:** Sample information for the species in the study.

Genus	Species	Abbreviations	Geographic region	Drainage	Partial morphological feature	
					Pairs of whiskers	Body scales
*Schizothorax*	*S. oconnori*	Soco	Gongga, Tibet, China	YarlungZangbo River	2	small scale
	*S. lissolabiatus*	Slis	Changdu, Tibet, China	Lancang River	2	small scale
	*S. nukiangensis*	Snuk	Bomi, Tibet, China	Nujiang River	2	small scale
	*S. plagiostomus*	Spla	Ali, Tibet, China	Shiquan River	2	small scale
	*S. labiatus*	Slab	Ali, Tibet, China	Shiquan River	2	small scale
	*S. davidi*	Sdav	Ganzi, Sichuan, China	Jinsha River	2	small scale
*Ptychobarbus*	*P. kaznakovi*	Pkaz	Changdu, Tibet, China	Lancang River	1	moderate degeneration
*Gymnocypris*	*G. namensis*	Gnam	Bange, Tibet, China	Lake Namtso	0	absence
	*G. przewalskii*	Gprz	Haibei, Qinghai, China	Lake Qinghai	0	absence
	*G. eckloni*	Geck	Xunhua, Qinghai, China	Yellow River	0	absence
	*G. selincuoensis*	Gsel	Bange, Tibet, China	Lake Siling Co	0	absence
*Schizopygopsis*	*S. younghusbandi*	Syou	Lazi, Tibet, China	YarlungZangbo River	0	absence
	*S. pylzovi*	Spyl	Xunhua, Qinghai, China	Yellow River	0	absence
*Platypharodon*	*P. extremus*	Pext	Gonghe, Qinghai, China	Yellow River	0	absence

**Table 2:** Sample collected for the transcriptome sequencing.

Species	The number of samples															
	Muscle	Liver	Spleen	Skin	Swim bladder	Gut	Eye	Gill	Kidney	Heart	Brain	Gonads	Vibrissa	Fat	Blood	Total
*S. oconnori*	1	1	1	1	1	1	1	1	1	1	1	1	1	1	1	15
*S. lissolabiatus*	1	1	1	1	1	1	1	1	1	1	1	1	1	—	1	14
*S. nukiangensis*	1	1	1	1	1	1	1	1	1	—	1	1	1	—	1	13
*S. plagiostomus*	1	1	1	1	1	1	1	1	1	1	1	—	—	—	1	12
*S. labiatus*	1	1	1	1	1	1	1	1	1	1	—	1	1	—	1	13
*S. davidi*	1	1	1	1	1	1	1	1	1	1	1	1	1	—	1	14
*P. kaznakovi*	1	1	1	1	—	1	1	1	1	1	1	1	1	—	1	13
*G. namensis*	1	1	1	1	1	1	1	1	1	1	1	1	—	—	1	13
*G. przewalskii*	1	1	1	1	1	1	1	1	1	1	1	1	—	1	—	13
*G. eckloni*	1	1	1	1	1	1	1	1	1	1	1	1	1	—	1	14
*G.selincuoensis*	1	1	1	—	1	1	1	1	1	1	1	1	—	1	1	13
*S. younghusbandi*	1	1	1	1	1	1	1	1	1	1	1	1	—	—	—	12
*S. pylzovi*	1	1	1	1	1	1	1	1	1	1	1	1	—	—	—	12
*P. extremus*	1	1	1	1	1	1	1	1	1	1	1	1	—	—	—	12
Total	14	14	14	13	13	14	14	14	14	13	13	13	7	3	10	183

The abbreviations of species were identical with those in Table 1. The short line represented the absence of the sample in the transcriptome sequencing.

## Methods

### Sample collection

To select representative Schizothoracine species in our study, we chose 14 species of 5 genera in Schizothoracine fishes representing the three specialized group based on the previous morphology study^5^. The primitive group in our study contains 6 species in *Schizothorax* genus, such as *Schizothorax oconnori* (*S. oconnori*), *Schizothorax lissolabiatus* (*S. lissolabiatus*), *Schizothorax nukiangensis* (*S. nukiangensis*), *Schizothorax plagiostomus* (*S. plagiostomus*), *Schizothorax labiatus* (*S. labiatus*) and *Schizothorax davidi* (*S. davidi*). The specialized group contains *Ptychobarbus kaznakovi* in *Ptychobarbus* genus. The highly specialized group contains 7 species in 3 genera, such as *Gymnocypris namensis* (*G. namensis*), *Gymnocypris przewalskii* (*G. przewalskii*), *Gymnocypris eckloni* (*G. eckloni*) and *Gymnocypris selincuoensis* (*G. selincuoensis*) of the *Gymnocypris* genus, *Schizopygopsis younghusbandi* (*S. younghusbandi*), and *Schizopygopsis pylzovi* (*S. pylzovi*) of the *Schizopygopsis* genus, *Platypharodon extremus* (*P. extremus*) in the *Platypharodon* genus. The samples were collected from the six major rivers and three lakes of the Tibetan Plateau including Yarlung Zangbo River, Shiquan River, Lancang River, Nujiang River, Jinsha River, Yellow River, Lake Namtso, Lake Qinghai, Lake Siling Co (Fig. 1 and Table 1). We noted that the Schizothoracine species in this work exhibited obvious morphology diversification, especially on the whiskers and scales. For example, *Gymnocypris, Schizopygopsis* and *Platypharodon* species were naked, while small scales were observed in the *Schizothorax* and *Ptychobarbus* genus (Table 1).

All individuals were narcotized with MS-222 (Solarbio, Beijing, China) for a few minutes before the sample collection. A total of 183 tissues were collected from 14 representative Schizothoracine fish in our study, including muscle, liver, spleen, gonads, skin, swim bladder, gut, eye, gill, kidney, heart, brain, blood, fat, vibrissa (Table 2). All tissues were immediately frozen in liquid nitrogen after the dissection and then stored at −80 °C until total RNA isolation.

### RNA extraction and sequencing

Total RNA was isolated from each sample using RNAiso Plus (TaKaRa, Dalian, China) according to the manufacturer’s instructions and was determined with a photometer for RNA sample integrity (Thermo Scientific, USA). RNA samples passing the quality criteria (see technical validation for detail) were used for the library preparation and RNA sequencing. All samples were sequenced on an Illumina HiSeq X Ten platform with 150 bp paired-end mode. In preset research, a total of more than 10 billion raw PE reads were obtained from all libraries. After filtering by removal of adaptor sequences, contaminated reads and poor–quality reads, we obtained approximately 1.4 Tb of clean data with Q20 bases larger than 96.94%. The average of 7.6 Gb sequencing data were obtained for samples (Supplementary Table S1). The transcriptome data for *Oxygymnocypris stewarti* in the *Oxygymnocypris* genus that reported in our previous studies^17^ were also used for comparision in the work.

### *De novo* assembly of fish transcriptome

**Fig. 2:**
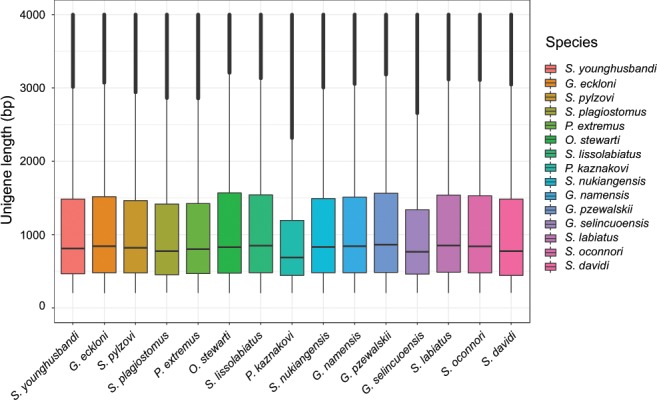
Length distribution of unigenes for all species.

We firstly utilized publicly available program Trinity software version 2.5.1^18^ with default parameters for *de novo* assembly of fish transcripts. The length of <200 bp contigs from each assembly libraries were discarded for subsequent analysis. Next, the redundancies of the transcripts for each species in the dataset were eliminated using the CD-HIT-EST program included in the cd-hit-v4.6.6 package^19^, with parameters -c 0.98 -n 11 -d 0 -M 0 -T 8 in the final assembly and the longest transcript in each cluster was considered as unigenes. After assembly, the unigene numbers for 15 Schizothoracine species ranged from 76,602 to 154,860 (Table 3). Of these, the highest number of unigenes was observed in *P. kaznakovi*, and the lowest in *S. labiatus*. The GC contents of transcripts for all species were rather stable around 40–42%. The N50 length of unigenes ranged from 1,564 to 2,143 bp, with an average of 1,250 bp for all fish transcriptome. As shown in Fig. 2, the unigene length distribution is comparable for all Schizothoracine species, and the average length ranged from 1,120 to 1,392 bp.

**Table 3:** The statistics of the *de novo* transcriptome assembly.

Species	Total size (Mb)	GC (%)	Unigene			Transcript		
			Sequence number	N50 length (bp)	Longest (bp)	Sequence number	N50 length (bp)	Longest (bp)
*S. oconnori*	117.00	0.415	88,676	1,948	36,581	831,353	1,527	36,694
*S. lissolabiatus*	104.06	0.422	79,073	1,946	33,187	667,802	1,573	33,187
*S. nukiangensis*	107.46	0.419	84,638	1,835	30,806	743,518	1,420	30,806
*S. plagiostomus*	98.95	0.419	83,169	1,725	17,902	736,405	1,255	17,910
*S. labiatus*	99.98	0.416	76,602	1,905	43,720	670,792	1,432	43,720
*S. davidi*	109.44	0.42	83,757	2,043	24,328	689,222	1,589	24,340
*P. kaznakovi*	173.48	0.409	154,860	1,564	77,434	1,363,461	1,198	77,434
*G. namensis*	107.09	0.415	84,464	1,825	23,933	813,474	1,294	23,933
*G. przewalskii*	105.49	0.413	78,762	1,974	28,230	751,137	1,409	28,231
*G. eckloni*	113.00	0.412	87,248	1,891	23,925	849,836	1,411	23,925
*G. selincuoensis*	122.36	0.406	106,851	1,588	25,730	1,187,251	914	25,730
*S. younghusbandi*	101.23	0.414	81,029	1,820	23,570	723,624	1,329	23,570
*S. pylzovi*	97.96	0.418	80,542	1,724	26,467	751,215	1,202	26,467
*P. extremus*	101.78	0.417	85,919	1,674	24,119	843,423	1,122	24,119
*O. stewartii*^#^	106.52	0.422	77,069	2,143	25,942	639,444	1,920	25,942

Note that the total size means the total base amount of all transcripts for species.

^#^The transcriptome data for *Oxygymnocypris stewarti* was reported in our previous studies^17^.

**Fig. 3:**
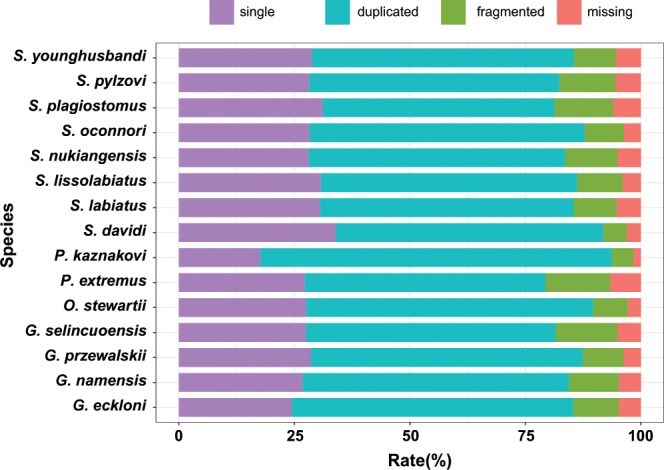
BUSCO statistics of assembled transcripts for species. The rate of single, duplicated, fragmented and missing BUSCO genes were colored by purple, blue, green and pink.

The assembled transcriptome sequences were analyzed by the BUSCO pipeline. BUSCO were generally used in the evaluation of the completeness of a genome assembly, we applied BUSCO version3.0.2 to assess the quality of transcriptome assembly in our work. As a result, we found that more than 98% of the 2,586 BUSCO genes of vertebrates were detected in our transcriptome and 85–92% were completely identified depends on species (Fig. 3), suggesting the transcriptome represented a rather high level of completeness of the conserved genes. Meanwhile, we found that a high fraction of duplicated BUSCO for all species (Fig. 3), which was consistent with the fact that the majority of the Schizothoracine fish were polyploidy.

### Functional annotation of transcriptome

To annotate the assembled unigenes, we searched the homologous sequences for all unigenes against four public available function databases (Blast-X search: E-value cutoff of 1 × 10^−10^), including NCBI nonredundant protein database (NR), Swiss-Prot, KEGG pathway database and KOG database. Only the best hits with the highest sequence homology was used for annotation. Then, the gene ontology (GO) terms analysis of the predicted protein based on the NR in NCBI was performed with the Blast2GO software version3.1 with default parameters. We found that at least 40.2% of unigenes per species were annotated based on proteins in four public databases (Table 4 and Supplementary Fig. S1). Meanwhile, we found that high match efficiency was observed the longer assembled unigenes (≥2,000 bp) compared to shorter unigenes (≤500 bp) during the annotation process, the same result was reported in other animal^20^.

**Table 4:** Functional annotation summary for species.

Species	NR	Swiss-port	KOG	GO	KEGG	Total	Ratio
*S. oconnori*	45,296	29,701	40,793	28,842	28,816	46,972	52.97%
*S. lissolabiatus*	45,091	30,793	41,064	30,203	29,922	46,516	58.83%
*S. nukiangensis*	46,557	31,077	42,380	30,450	30,185	48,122	56.86%
*S. plagiostomus*	49,111	33,194	44,034	34,896	32,267	51,264	61.64%
*S. labiatus*	43,749	29,702	39,846	28,668	28,837	44,956	58.69%
*S. davidi*	47,898	32,467	42,544	35,628	31,610	50,962	60.85%
*P. kaznakovi*	58,392	34,174	49,960	33,669	33,253	62,216	40.18%
*G. namensis*	44,310	29,970	40,147	28,721	29,102	45,732	54.14%
*G. przewalskii*	43,104	29,502	39,141	28,524	28,628	44,387	56.36%
*G. eckloni*	45,847	31,699	41,648	30,754	30,813	47,353	54.27%
*G. selincuoensis*	49,768	32,165	44,381	31,049	31,239	51,828	48.50%
*S. younghusbandi*	46,369	33,008	42,487	31,612	32,070	47,533	58.66%
*S. pylzovi*	44,777	31,296	41,101	30,088	30,408	46,094	57.23%
*P. extremus*	46,694	32,136	42,756	30,766	31,231	48,074	55.95%
*O. stewartii*^#^	43,212	29,426	38,495	32,099	28,597	46,009	59.70%

The hit number for NR, Swiss-port, KOG, GO, KEGG were summarized. The ratio means the percentage of annotated unigenes to the total assembly sequences.

^#^The transcriptome data for *Oxygymnocypris stewarti* was reported in our previous studies^17^.

## Data Records

The sequencing and assembly data of transcriptome for all samples were deposited into public repositories: The transcriptome sequencing data generated in this work were deposited as SRP186751 in NCBI Sequence Read Archive^21^; The assembly of sequencing data were deposited in TSA as GHYM00000000^22^, GHYL00000000^23^, GHYK00000000^24^, GHYJ00000000^25^, GHYI00000000^26^, GHYH00000000^27^, GHYG00000000^28^, GHYF00000000^29^, GHYE00000000^30^, GHYD00000000^31^, GHYC00000000^32^, GHYB00000000^33^, GHYA00000000^34^, GIBO00000000^35^, and GHXZ00000000^36^; The transcriptome annotation information and predicted coding and protein sequences for unigenes were uploaded to figshare^37^.

## Technical Validation

### RNA integrity

The transcriptome for twelve tissues from three fish individuals were sequenced. In before constructing RNA-Seq libraries, the concentration and quality of total RNA were evaluated using NanoVue Plus spectrophotometer (GE Healthcare, NJ, USA). The total amount of RNA, RNA integrity and rRNA ratio were used to estimate the quality, content and degradation level of RNA samples. In the present study, RNAs samples with a total RNA amount ≥ 10 μg, RNA integrity number ≥ 8, and rRNA ratio ≥ 1.5 were finally subjected to construct the sequencing library.

### Quality filtering of Illumina sequencing raw reads

The raw sequencing reads generated from the Illumina platform were rigorously cleaned by the following procedures as in the previous study^38^. Firstly, adaptors in the reads were filtered out; secondly, reads with more than 10% of N bases were filtered out; thirdly, reads with more than 50% of the low-quality bases (phred quality score < =5) were filtered out. If any end of the pair was classified as low quality, both pairs were discarded. The initially generated raw sequencing reads were also evaluated regarding quality distribution, GC content distribution, base composition, average quality score at each position and other metrics.

## Supplementary information

Supplementary Table S1

Supplementary Figure S1

## Data Availability

No specific code or script was used in this work. All commands used in the data processing were executed as the manual and usage instrument of the corresponding bioinformatics software.
