# Supplementary material for: Comprehensive transcriptome data for endemic Schizothoracinae fish in the Tibetan Plateau
Source: Sci Data. 2020 Jan 21;7:28. doi: 10.1038/s41597-020-0361-6 (PMC6972879; doi:10.1038/s41597-020-0361-6)
Supplement: Supplementary file 1 — Supplementary Table S1 [file 41597_2020_361_MOESM1_ESM.pdf]

**Supplementary Table S1 Sequencing output for samples.**

| Sample | Raw Reads | Clean Reads | Clean Bases | Error rate | Q20 | Q30 | GC content |
|---|---|---|---|---|---|---|---|
| ***Gymnocypris eckloni*** | | | | | | | |
| **Muscle** | 4.7E+07 | 4.5E+07 | 6.76G | 0.03 | 96.78 | 91.92 | 47.18 |
| **Liver** | 5.8E+07 | 5.6E+07 | 8.36G | 0.03 | 96.86 | 92.12 | 45.56 |
| **Spleen** | 4.6E+07 | 4.5E+07 | 6.74G | 0.03 | 96.65 | 91.74 | 44.59 |
| **Skin** | 6.4E+07 | 6.2E+07 | 9.24G | 0.03 | 96.74 | 91.95 | 45.09 |
| **Swim bladder** | 5.5E+07 | 5.2E+07 | 7.86G | 0.02 | 97.88 | 94.39 | 44.63 |
| **Gut** | 1E+08 | 9.9E+07 | 14.80G | 0.03 | 96.65 | 91.70 | 44.85 |
| **Eye** | 6.2E+07 | 6E+07 | 9.04G | 0.03 | 96.58 | 91.70 | 43.13 |
| **Gill** | 4.8E+07 | 4.6E+07 | 6.90G | 0.03 | 96.39 | 91.15 | 45.20 |
| **Kidney** | 5.1E+07 | 4.9E+07 | 7.40G | 0.03 | 96.47 | 91.48 | 42.99 |
| **Heart** | 5.9E+07 | 5.7E+07 | 8.52G | 0.03 | 96.72 | 91.88 | 44.32 |
| **Brain** | 5.6E+07 | 5.3E+07 | 8.02G | 0.03 | 95.69 | 89.83 | 44.13 |
| **Gonads** | 6.5E+07 | 6.2E+07 | 9.32G | 0.03 | 96.50 | 91.57 | 47.71 |
| **Fat** | 5E+07 | 4.7E+07 | 7.10G | 0.03 | 96.96 | 92.16 | 50.40 |
| **Blood** | 4.7E+07 | 4.6E+07 | 6.86G | 0.03 | 96.63 | 91.74 | 44.56 |
| ***Gymnocypris selincuoensis*** | | | | | | | |
| **Sample** | **Raw Reads** | **Clean Reads** | **Clean Bases** | **Error rate** | **Q20** | **Q30** | **GC content** |
| **Muscle** | 6.1E+07 | 5.9E+07 | 8.86G | 0.02 | 97.95 | 94.74 | 47.52 |
| **Liver** | 5E+07 | 4.8E+07 | 7.22G | 0.02 | 97.42 | 93.30 | 46.59 |
| **Spleen** | 5.8E+07 | 5.6E+07 | 8.42G | 0.02 | 98.03 | 94.88 | 46.92 |
| **Swim bladder** | 5.2E+07 | 5E+07 | 7.52G | 0.02 | 97.98 | 94.79 | 47.52 |
| **Gut** | 5.9E+07 | 5.6E+07 | 8.46G | 0.02 | 98.02 | 94.86 | 46.09 |
| **Eye** | 5.7E+07 | 5.5E+07 | 8.24G | 0.02 | 98.10 | 95.06 | 45.73 |
| **Gill** | 5.6E+07 | 5.4E+07 | 8.09G | 0.02 | 98.08 | 94.99 | 46.68 |
| **Kidney** | 5.8E+07 | 5.6E+07 | 8.43G | 0.02 | 98.12 | 95.06 | 46.72 |
| **Heart** | 5.4E+07 | 5.2E+07 | 7.86G | 0.02 | 97.98 | 94.82 | 46.43 |
| **Brain** | 5.1E+07 | 4.9E+07 | 7.40G | 0.02 | 98.00 | 94.86 | 45.79 |
| **Gonads** | 5.5E+07 | 5.2E+07 | 7.84G | 0.02 | 97.68 | 94.39 | 48.07 |
| **Fat** | 4.7E+07 | 4.5E+07 | 6.82G | 0.02 | 98.29 | 95.33 | 49.66 |
| **Blood** | 5.2E+07 | 5E+07 | 7.46G | 0.02 | 98.21 | 95.21 | 48.45 |
| ***Schizopygopsis younghusbandi*** | | | | | | | |
| **Sample** | **Raw Reads** | **Clean Reads** | **Clean Bases** | **Error rate** | **Q20** | **Q30** | **GC content** |
| **Muscle** | 5.3E+07 | 5.1E+07 | 7.68G | 0.02 | 96.88 | 92.15 | 47.86 |
| **Liver** | 5.2E+07 | 5E+07 | 7.49G | 0.02 | 97.02 | 92.45 | 46.85 |
| **Spleen** | 4.3E+07 | 4.1E+07 | 6.14G | 0.02 | 97.12 | 92.53 | 50.80 |
| **Skin** | 4.3E+07 | 4.1E+07 | 6.20G | 0.02 | 96.84 | 92.00 | 46.84 |
| **Swim bladder** | 4.5E+07 | 4.3E+07 | 6.52G | 0.02 | 96.88 | 92.06 | 46.64 |
| **Gut** | 5.1E+07 | 4.8E+07 | 7.25G | 0.02 | 96.96 | 92.26 | 46.03 |
| **Eye** | 5.2E+07 | 5E+07 | 7.43G | 0.02 | 96.61 | 91.54 | 46.65 |
| **Gill** | 5E+07 | 4.8E+07 | 7.24G | 0.02 | 96.80 | 91.98 | 45.59 |
| **Kidney** | 4.4E+07 | 4.2E+07 | 6.34G | 0.02 | 96.83 | 92.00 | 46.10 |
| **Heart** | 5.2E+07 | 4.9E+07 | 7.42G | 0.02 | 96.81 | 91.98 | 45.96 |

| | | | | | | | |
|---|---|---|---|---|---|---|---|
| **Brain** | 5E+07 | 4.8E+07 | 7.19G | 0.01 | 97.36 | 93.24 | 44.13 |
| **Gonads** | 4.6E+07 | 4.4E+07 | 6.62G | 0.02 | 96.76 | 92.18 | 46.92 |
| ***Schizopygopsis pylzovi*** | | | | | | | |
| **Sample** | **Raw Reads** | **Clean Reads** | **Clean Bases** | **Error rate** | **Q20** | **Q30** | **GC content** |
| **Muscle** | 4.8E+07 | 4.5E+07 | 6.73G | 0.01 | 97.66 | 93.87 | 49.23 |
| **Liver** | 4.3E+07 | 4.3E+07 | 6.48G | 0.01 | 97.73 | 94.06 | 47.79 |
| **Spleen** | 4.6E+07 | 4.3E+07 | 6.49G | 0.01 | 97.39 | 93.37 | 47.84 |
| **Skin** | 4.9E+07 | 4.6E+07 | 6.95G | 0.01 | 97.39 | 93.33 | 48.77 |
| **Swim bladder** | 4.6E+07 | 4.3E+07 | 6.45G | 0.01 | 97.33 | 93.21 | 48.83 |
| **Gut** | 4.4E+07 | 4.3E+07 | 6.13G | 0.01 | 97.43 | 93.49 | 46.88 |
| **Eye** | 5E+07 | 4.7E+07 | 7.01G | 0.01 | 97.50 | 93.56 | 48.81 |
| **Gill** | 5.3E+07 | 4.9E+07 | 7.39G | 0.01 | 97.55 | 93.68 | 47.54 |
| **Kidney** | 5.1E+07 | 4.8E+07 | 7.25G | 0.01 | 97.44 | 93.44 | 47.31 |
| **Heart** | 4.3E+07 | 4E+07 | 5.93G | 0.01 | 97.50 | 93.59 | 47.33 |
| **Brain** | 5E+07 | 4.7E+07 | 7.08G | 0.01 | 97.30 | 93.22 | 45.34 |
| **Gonads** | 5E+07 | 3.6E+07 | 5.45G | 0.01 | 97.18 | 93.09 | 48.20 |
| ***Platypharodon extremus*** | | | | | | | |
| **Sample** | **Raw Reads** | **Clean Reads** | **Clean Bases** | **Error rate** | **Q20** | **Q30** | **GC content** |
| **Muscle** | 4.5E+07 | 4.3E+07 | 6.40G | 0.01 | 97.97 | 94.64 | 47.96 |
| **Liver** | 5.5E+07 | 5.2E+07 | 7.83G | 0.01 | 97.96 | 94.66 | 46.72 |
| **Spleen** | 5.1E+07 | 4.9E+07 | 7.28G | 0.01 | 97.83 | 94.40 | 47.55 |
| **Skin** | 4.8E+07 | 4.6E+07 | 6.86G | 0.01 | 97.82 | 94.36 | 48.99 |
| **Swim bladder** | 4.7E+07 | 4.5E+07 | 6.78G | 0.01 | 97.82 | 94.32 | 49.06 |
| **Gut** | 4.5E+07 | 4.3E+07 | 6.45G | 0.01 | 97.96 | 94.65 | 47.54 |
| **Eye** | 4.3E+07 | 4.2E+07 | 6.28G | 0.02 | 96.87 | 92.34 | 48.91 |
| **Gill** | 5.4E+07 | 5.1E+07 | 7.71G | 0.01 | 97.81 | 94.39 | 47.45 |
| **Kidney** | 5E+07 | 4.8E+07 | 7.18G | 0.01 | 97.74 | 94.24 | 47.25 |
| **Heart** | 4.6E+07 | 4.4E+07 | 6.57G | 0.01 | 97.80 | 94.35 | 48.26 |
| **Brain** | 5.7E+07 | 5.4E+07 | 8.10G | 0.01 | 97.78 | 94.32 | 45.66 |
| **Gonads** | 5.2E+07 | 4.9E+07 | 7.37G | 0.01 | 97.83 | 94.33 | 49.92 |
| ***Schizothorax oconnori*** | | | | | | | |
| **Sample** | **Raw Reads** | **Clean Reads** | **Clean Bases** | **Error rate** | **Q20** | **Q30** | **GC content** |
| **Muscle** | 6.2E+07 | 6E+07 | 9.08G | 0.03 | 96.56 | 91.50 | 47.91 |
| **Liver** | 7E+07 | 6.8E+07 | 10.16G | 0.03 | 96.84 | 92.06 | 46.41 |
| **Spleen** | 6.6E+07 | 6.4E+07 | 9.56G | 0.03 | 96.69 | 91.82 | 46.08 |
| **Skin** | 4.4E+07 | 4.3E+07 | 6.44G | 0.03 | 96.32 | 91.00 | 47.19 |
| **Swim bladder** | 5.2E+07 | 5.1E+07 | 7.62G | 0.03 | 96.06 | 90.46 | 46.97 |
| **Gut** | 6.2E+07 | 6E+07 | 9.06G | 0.03 | 96.57 | 91.57 | 45.95 |
| **Eye** | 5.3E+07 | 5.2E+07 | 7.76G | 0.03 | 96.61 | 91.62 | 46.20 |
| **Gill** | 5.4E+07 | 5.2E+07 | 7.86G | 0.03 | 96.55 | 91.52 | 45.78 |
| **Kidney** | 5.2E+07 | 5.1E+07 | 7.60G | 0.03 | 96.39 | 91.17 | 45.90 |
| **Heart** | 6.1E+07 | 5.9E+07 | 8.84G | 0.03 | 96.75 | 91.93 | 45.86 |
| **Brain** | 5.7E+07 | 5.5E+07 | 8.18G | 0.03 | 96.57 | 91.57 | 44.60 |
| **Gonads** | 6.2E+07 | 5.9E+07 | 8.86G | 0.04 | 94.97 | 88.56 | 48.12 |
| **vibrissa** | 7.3E+07 | 6.9E+07 | 10.40G | 0.03 | 96.16 | 90.76 | 45.55 |

| | | | | | | | |
|---|---|---|---|---|---|---|---|
| **Fat** | 6.1E+07 | 6E+07 | 8.96G | 0.03 | 96.98 | 92.28 | 50.19 |
| **Blood** | 4.5E+07 | 4.3E+07 | 6.50G | 0.03 | 96.32 | 91.02 | 45.71 |
| ***Schizothorax lissolabiatus*** | | | | | | | |
| **Sample** | **Raw Reads** | **Clean Reads** | **Clean Bases** | **Error rate** | **Q20** | **Q30** | **GC content** |
| **Muscle** | 4.5E+07 | 4.3E+07 | 6.44G | 0.02 | 97.28 | 93.53 | 49.89 |
| **Liver** | 4.3E+07 | 4.2E+07 | 6.34G | 0.03 | 97.38 | 93.38 | 47.65 |
| **Spleen** | 6.9E+07 | 6.6E+07 | 9.96G | 0.03 | 96.68 | 92.35 | 47.72 |
| **Skin** | 5.3E+07 | 5E+07 | 7.56G | 0.03 | 96.81 | 92.67 | 49.73 |
| **Swim bladder** | 5.8E+07 | 5.5E+07 | 8.32G | 0.03 | 96.05 | 91.46 | 49.18 |
| **Gut** | 5.4E+07 | 5.2E+07 | 7.84G | 0.03 | 97.08 | 93.15 | 47.18 |
| **Eye** | 4.1E+07 | 3.9E+07 | 5.90G | 0.02 | 97.23 | 93.41 | 48.87 |
| **Gill** | 4.5E+07 | 4.4E+07 | 6.54G | 0.02 | 97.31 | 93.34 | 47.32 |
| **Kidney** | 4.4E+07 | 4.3E+07 | 6.48G | 0.02 | 97.41 | 93.48 | 47.52 |
| **Heart** | 5E+07 | 4.8E+07 | 7.20G | 0.02 | 97.19 | 93.40 | 49.22 |
| **Brain** | 5E+07 | 4.8E+07 | 7.26G | 0.03 | 97.10 | 93.18 | 46.94 |
| **Gonads** | 4.6E+07 | 4.5E+07 | 6.68G | 0.02 | 97.26 | 93.06 | 48.44 |
| **vibrissa** | 5.4E+07 | 5.2E+07 | 7.74G | 0.03 | 97.08 | 93.16 | 48.59 |
| **Blood** | 5.1E+07 | 4.9E+07 | 7.32G | 0.03 | 96.73 | 92.05 | 49.61 |
| ***Schizothorax nukiangensis*** | | | | | | | |
| **Sample** | **Raw Reads** | **Clean Reads** | **Clean Bases** | **Error rate** | **Q20** | **Q30** | **GC content** |
| **Muscle** | 4.4E+07 | 4.3E+07 | 6.48G | 0.03 | 97.10 | 92.61 | 48.61 |
| **Liver** | 4.5E+07 | 4.3E+07 | 6.46G | 0.02 | 97.39 | 93.78 | 47.66 |
| **Spleen** | 4.1E+07 | 3.9E+07 | 5.88G | 0.02 | 97.26 | 93.50 | 47.65 |
| **Skin** | 3.9E+07 | 3.8E+07 | 5.72G | 0.02 | 97.37 | 93.28 | 48.22 |
| **Swim bladder** | 5.3E+07 | 5E+07 | 7.50G | 0.03 | 97.10 | 93.16 | 49.30 |
| **Gut** | 6E+07 | 5.7E+07 | 8.60G | 0.02 | 97.24 | 93.45 | 48.59 |
| **Eye** | 5.4E+07 | 5.1E+07 | 7.70G | 0.03 | 96.94 | 92.86 | 47.58 |
| **Gill** | 5.5E+07 | 5.3E+07 | 7.94G | 0.03 | 97.07 | 93.19 | 47.43 |
| **Kidney** | 5.1E+07 | 4.9E+07 | 7.30G | 0.03 | 97.14 | 93.27 | 47.62 |
| **Brain** | 4.7E+07 | 4.5E+07 | 6.78G | 0.03 | 97.07 | 93.15 | 46.30 |
| **Gonads** | 3.8E+07 | 3.6E+07 | 5.48G | 0.03 | 97.25 | 92.97 | 46.56 |
| **vibrissa** | 5.6E+07 | 5.4E+07 | 8.06G | 0.03 | 96.76 | 92.15 | 47.19 |
| **Blood** | 4.5E+07 | 4.4E+07 | 6.58G | 0.03 | 96.67 | 91.97 | 46.51 |
| ***Schizothorax plagiostomus*** | | | | | | | |
| **Sample** | **Raw Reads** | **Clean Reads** | **Clean Bases** | **Error rate** | **Q20** | **Q30** | **GC content** |
| **Muscle** | 4.6E+07 | 4.5E+07 | 6.72G | 0.03 | 96.51 | 91.56 | 48.93 |
| **Liver** | 3.5E+07 | 3.4E+07 | 5.08G | 0.03 | 96.57 | 91.69 | 46.37 |
| **Spleen** | 4.7E+07 | 4.6E+07 | 6.82G | 0.03 | 96.44 | 91.44 | 49.39 |
| **Swim bladder** | 4.3E+07 | 4.2E+07 | 6.24G | 0.03 | 96.42 | 91.42 | 46.19 |
| **Gut** | 3.8E+07 | 3.7E+07 | 5.50G | 0.03 | 96.40 | 91.44 | 44.85 |
| **Eye** | 4.3E+07 | 4.2E+07 | 6.26G | 0.03 | 96.29 | 91.22 | 45.85 |
| **Brain** | 4.2E+07 | 4.1E+07 | 6.16G | 0.03 | 96.04 | 90.81 | 44.00 |
| **Gill** | 5.2E+07 | 5E+07 | 7.49G | 0.03 | 97.36 | 93.16 | 46.25 |
| **Kidney** | 4.6E+07 | 4.5E+07 | 6.68G | 0.03 | 97.41 | 93.25 | 46.45 |
| **Heart** | 5.8E+07 | 5.6E+07 | 8.45G | 0.03 | 97.47 | 93.37 | 46.66 |

| | | | | | | | |
|---|---|---|---|---|---|---|---|
| **Brain** | 4.2E+07 | 4.1E+07 | 6.16G | 0.03 | 96.04 | 90.80 | 44.00 |
| **Blood** | 5.6E+07 | 5.4E+07 | 8.05G | 0.03 | 97.31 | 93.09 | 47.86 |
| ***Schizothorax labiatus*** | | | | | | | |
| **Sample** | **Raw Reads** | **Clean Reads** | **Clean Bases** | **Error rate** | **Q20** | **Q30** | **GC content** |
| **Muscle** | 5.4E+07 | 5.2E+07 | 7.77G | 0.01 | 97.41 | 93.19 | 47.83 |
| **Liver** | 5E+07 | 4.8E+07 | 7.25G | 0.02 | 97.28 | 92.96 | 46.65 |
| **Spleen** | 5.4E+07 | 5.2E+07 | 7.85G | 0.02 | 97.28 | 93.02 | 45.99 |
| **Skin** | 4.8E+07 | 4.6E+07 | 6.84G | 0.02 | 97.22 | 92.90 | 47.05 |
| **Swim bladder** | 4.6E+07 | 4.4E+07 | 6.54G | 0.02 | 97.22 | 92.87 | 46.71 |
| **Gut** | 5.1E+07 | 4.9E+07 | 7.33G | 0.02 | 96.98 | 92.43 | 46.05 |
| **Eye** | 4.7E+07 | 4.5E+07 | 6.79G | 0.01 | 97.29 | 93.05 | 46.91 |
| **Gill** | 4.3E+07 | 4.1E+07 | 6.21G | 0.01 | 97.30 | 93.03 | 46.46 |
| **Kidney** | 5.5E+07 | 5.3E+07 | 8.00G | 0.01 | 97.39 | 93.24 | 46.14 |
| **Heart** | 5.2E+07 | 5E+07 | 7.54G | 0.02 | 96.63 | 91.54 | 46.28 |
| **Gonads** | 4.9E+07 | 4.7E+07 | 7.04G | 0.02 | 96.28 | 91.12 | 47.52 |
| **vibrissa** | 5.2E+07 | 5E+07 | 7.51G | 0.02 | 97.12 | 92.70 | 47.42 |
| **Blood** | 4.3E+07 | 4.1E+07 | 6.22G | 0.01 | 97.38 | 93.17 | 48.70 |
| ***Schizothorax davidi*** | | | | | | | |
| **Sample** | **Raw Reads** | **Clean Reads** | **Clean Bases** | **Error rate** | **Q20** | **Q30** | **GC content** |
| **Muscle** | 5E+07 | 4.9E+07 | 7.41G | 0.02 | 97.04 | 92.19 | 48.37 |
| **Liver** | 4.6E+07 | 4.6E+07 | 6.90G | 0.02 | 96.93 | 91.83 | 47.04 |
| **Spleen** | 4.9E+07 | 4.8E+07 | 7.27G | 0.02 | 97.07 | 92.19 | 46.50 |
| **Skin** | 4.6E+07 | 4.5E+07 | 6.74G | 0.02 | 97.30 | 92.89 | 50.54 |
| **Swim bladder** | 5.2E+07 | 5.1E+07 | 7.69G | 0.02 | 96.98 | 92.02 | 46.49 |
| **Gut** | 5.1E+07 | 5.1E+07 | 7.63G | 0.02 | 97.95 | 94.11 | 46.66 |
| **Eye** | 5E+07 | 4.9E+07 | 7.42G | 0.02 | 97.34 | 92.93 | 47.03 |
| **Gill** | 5E+07 | 4.9E+07 | 7.4G | 0.02 | 96.94 | 91.98 | 46.33 |
| **Kidney** | 4.8E+07 | 4.7E+07 | 7.07G | 0.02 | 97.09 | 92.27 | 47.02 |
| **Heart** | 4.8E+07 | 4.7E+07 | 7.10G | 0.02 | 97.03 | 92.09 | 46.93 |
| **Brain** | 5E+07 | 5E+07 | 7.43G | 0.02 | 97.16 | 92.41 | 45.61 |
| **Gonads** | 4.9E+07 | 4.9E+07 | 7.29G | 0.02 | 97.06 | 92.19 | 46.85 |
| **vibrissa** | 4.9E+07 | 4.8E+07 | 7.24G | 0.02 | 95.41 | 88.06 | 47.14 |
| **Blood** | 4.6E+07 | 4.6E+07 | 6.83G | 0.02 | 97.50 | 93.25 | 50.72 |
| ***Ptychobarbus kaznakovi*** | | | | | | | |
| **Sample** | **Raw Reads** | **Clean Reads** | **Clean Bases** | **Error rate** | **Q20** | **Q30** | **GC content** |
| **Muscle** | 5E+07 | 4.8E+07 | 7.18G | 0.03 | 96.67 | 91.94 | 49.27 |
| **Liver** | 4.9E+07 | 4.7E+07 | 7.02G | 0.03 | 96.56 | 91.81 | 47.60 |
| **Spleen** | 5.3E+07 | 5.1E+07 | 7.60G | 0.03 | 96.74 | 92.16 | 47.24 |
| **Skin** | 4.9E+07 | 4.8E+07 | 7.14G | 0.03 | 96.31 | 91.32 | 48.09 |
| **Gut** | 6.1E+07 | 5.8E+07 | 8.76G | 0.03 | 96.57 | 91.78 | 47.09 |
| **Eye** | 4.9E+07 | 4.7E+07 | 7.06G | 0.03 | 96.66 | 91.97 | 47.03 |
| **Gill** | 5.6E+07 | 5.4E+07 | 8.16G | 0.03 | 96.46 | 91.63 | 46.41 |
| **Kidney** | 4.3E+07 | 3.8E+07 | 5.76G | 0.03 | 95.91 | 90.58 | 46.70 |
| **Heart** | 4.4E+07 | 4.3E+07 | 6.42G | 0.03 | 95.65 | 89.73 | 47.03 |
| **Brain** | 5.1E+07 | 4.9E+07 | 7.32G | 0.03 | 96.53 | 91.73 | 46.23 |
| **Gonads** | 5.5E+07 | 5.3E+07 | 7.98G | 0.03 | 97.15 | 92.79 | 46.50 |
| **vibrissa** | 4.9E+07 | 4.7E+07 | 7.10G | 0.03 | 96.35 | 91.39 | 47.04 |

| | | | | | | | |
|---|---|---|---|---|---|---|---|
| **Blood** | 6.5E+07 | 6.2E+07 | 9.23G | 0.03 | 95.39 | 89.66 | 49.05 |
| ***Gymnocypris namensis*** | | | | | | | |
| **Sample** | **Raw Reads** | **Clean Reads** | **Clean Bases** | **Error rate** | **Q20** | **Q30** | **GC content** |
| **Muscle** | 4.9E+07 | 4.7E+07 | 7.06G | 0.03 | 96.44 | 91.46 | 49.20 |
| **Liver** | 6E+07 | 5.7E+07 | 8.56G | 0.03 | 96.74 | 92.11 | 47.77 |
| **Spleen** | 5.7E+07 | 5.5E+07 | 8.30G | 0.02 | 97.47 | 93.67 | 47.46 |
| **Skin** | 5E+07 | 4.7E+07 | 7.10G | 0.03 | 96.53 | 91.71 | 48.01 |
| **Swim bladder** | 4.6E+07 | 4.4E+07 | 6.56G | 0.03 | 96.85 | 92.28 | 47.22 |
| **Gut** | 4.3E+07 | 4.1E+07 | 6.20G | 0.03 | 96.86 | 92.34 | 46.80 |
| **Eye** | 5.9E+07 | 5.6E+07 | 8.42G | 0.03 | 96.59 | 91.84 | 47.14 |
| **Gill** | 5.4E+07 | 5.2E+07 | 7.84G | 0.03 | 96.73 | 92.13 | 46.30 |
| **Kidney** | 5E+07 | 4.8E+07 | 7.16G | 0.03 | 96.97 | 92.56 | 46.83 |
| **Heart** | 5.3E+07 | 5.1E+07 | 7.60G | 0.03 | 96.51 | 91.68 | 47.87 |
| **Brain** | 5E+07 | 4.8E+07 | 7.16G | 0.03 | 96.56 | 91.79 | 46.64 |
| **Gonads** | 5.9E+07 | 5.6E+07 | 8.46G | 0.03 | 96.15 | 91.18 | 48.79 |
| **Blood** | 4.9E+07 | 4.7E+07 | 7.12G | 0.03 | 96.57 | 91.74 | 48.39 |
| ***Gymnocypris przewalskii*** | | | | | | | |
| **Sample** | **Raw Reads** | **Clean Reads** | **Clean Bases** | **Error rate** | **Q20** | **Q30** | **GC content** |
| **Muscle** | 5.6E+07 | 5.5E+07 | 8.18G | 0.03 | 96.84 | 92.04 | 46.01 |
| **Liver** | 5.3E+07 | 5.2E+07 | 7.78G | 0.03 | 96.90 | 92.15 | 46.11 |
| **Spleen** | 4.5E+07 | 4.4E+07 | 6.56G | 0.03 | 96.91 | 92.19 | 46.27 |
| **Skin** | 4.8E+07 | 4.7E+07 | 7.00G | 0.03 | 96.73 | 91.89 | 46.60 |
| **Swim bladder** | 5.8E+07 | 5.7E+07 | 8.48G | 0.03 | 96.52 | 91.47 | 46.86 |
| **Gut** | 5E+07 | 4.8E+07 | 7.26G | 0.03 | 96.87 | 92.09 | 46.15 |
| **Eye** | 6.8E+07 | 6.6E+07 | 9.94G | 0.03 | 96.91 | 92.27 | 46.33 |
| **Gill** | 5.4E+07 | 5.2E+07 | 7.82G | 0.03 | 96.88 | 92.20 | 45.62 |
| **Kidney** | 5.9E+07 | 5.7E+07 | 8.54G | 0.03 | 96.76 | 91.89 | 45.84 |
| **Heart** | 5.3E+07 | 5.1E+07 | 7.62G | 0.03 | 95.72 | 89.80 | 45.84 |
| **Brain** | 5.9E+07 | 5.7E+07 | 8.58G | 0.03 | 96.70 | 91.81 | 44.97 |
| **Gonads** | 4.7E+07 | 4.5E+07 | 6.78G | 0.03 | 96.39 | 91.30 | 48.02 |
| **Fat** | 5.1E+07 | 4.9E+07 | 7.40G | 0.03 | 97.03 | 92.26 | 50.29 |
