# Supplementary figures and images for: Comprehensive transcriptome data for endemic Schizothoracinae fish in the Tibetan Plateau
Source: Sci Data. 2020 Jan 21;7:28. doi: 10.1038/s41597-020-0361-6 (PMC6972879; doi:10.1038/s41597-020-0361-6)

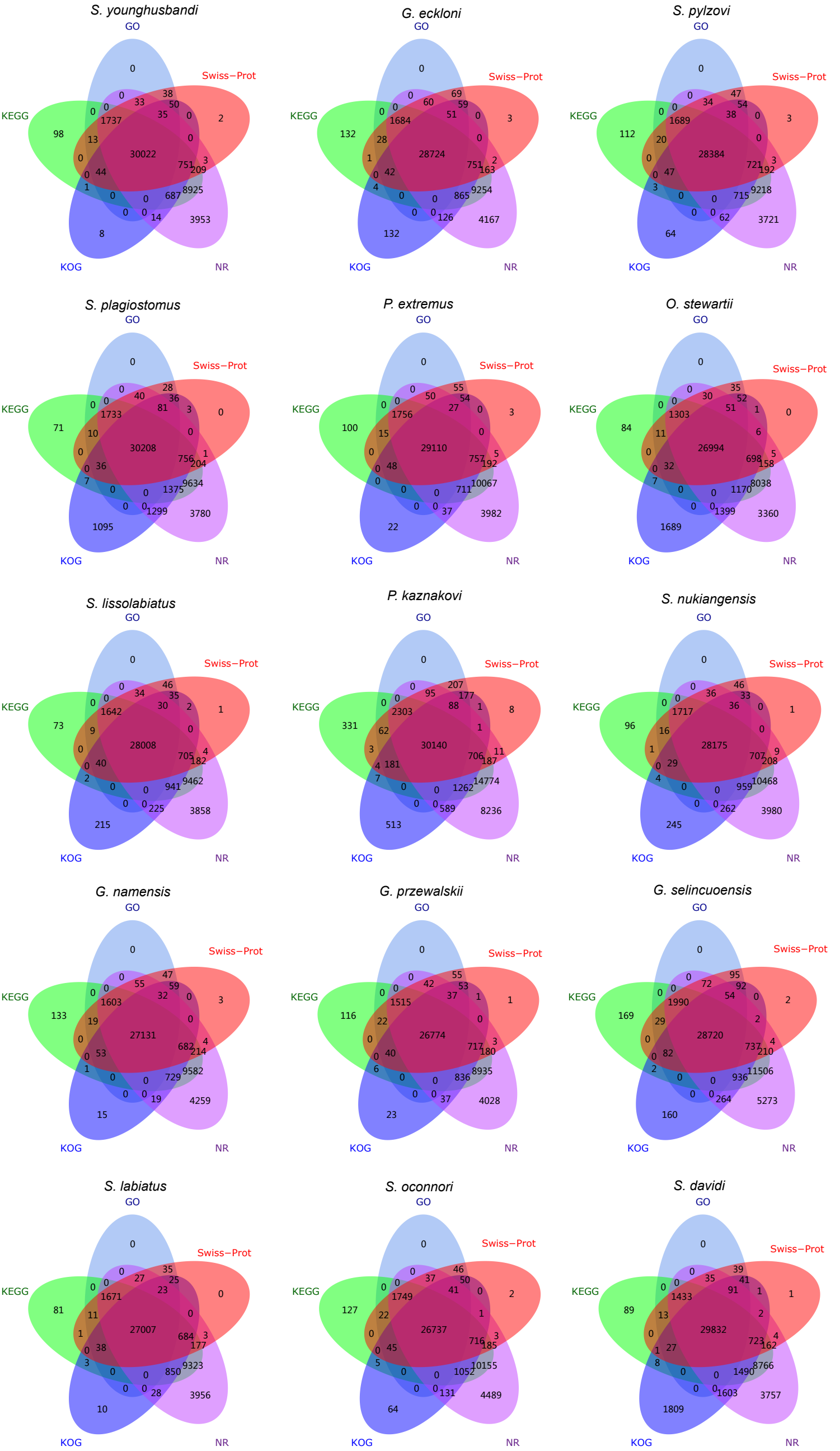

**Supplementary Figure S1 Venn plot for the functional annotations.**

Supplement: Supplementary file 2 — Supplementary Figure S1 [file 41597_2020_361_MOESM2_ESM.pdf]
